# Aspects of self-regulated learning and their influence on the mathematics achievement of fifth graders in the context of four different proclaimed curricula

**DOI:** 10.3389/fpsyg.2022.963151

**Published:** 2022-10-11

**Authors:** Jaroslav Říčan, Vlastimil Chytrý, Janka Medová

**Affiliations:** ^1^Department of Education and Applied Disciplines, Faculty of Education, Jan Evangelista Purkyně University in Ústí nad Labem, Ústí nad Labem, Czechia; ^2^Department of Preschool and Primary Education, Faculty of Education, Jan Evangelista Purkyně University in Ústí nad Labem, Ústí nad Labem, Czechia; ^3^Department of Mathematics, Faculty of Natural Sciences and Informatics, Constantine the Philosopher University in Nitra, Nitra, Slovakia

**Keywords:** metacognitive knowledge, metacognitive monitoring, mathematical self-efficacy, mathematics achievement, confidence judgments

## Abstract

Metacognition is a part of the models of self-regulated learning. The consideration of a broader context resonates with a social cognitive perspective approach to learning which dominates the educational academic field with the theory of self-regulated learning. Metacognition is considered a crucial factor influencing mathematics achievement. Furthermore, the affective field including pupils' self-efficacy, interest and motivation are the phenomena involved in mathematical problem-solving. On the other hand, metacognitive knowledge and metacognitive regulations are not a regular part of mathematics education in the Czech Republic. The main aim of this study was to investigate the relation between pupils' attitude toward mathematics; metacognitive knowledge; self-efficacy and motivation; metacognitive monitoring; and their achievement in solving mathematical problems. All together 1,133 students of Grade 5 from four types of Czech schools participated in the study. There were traditional schools; schools teaching mathematics by genetic constructivism, i.e., Hejný's method; Montessori schools; and Dalton schools were involved. The assessed variables, namely relation to mathematics; metacognitive knowledge; self-efficacy and motivation; metacognitive monitoring; and mathematical achievement were used as an input to regression analysis. Item-response theory was used for assessing the performance of the students and demands of the tasks. The metacognitive monitoring was detected as the most significant predictor of mathematics achievement for higher- and lower-performing students as well as for the item with high and low demands. The study reveals how the different mathematics curricula (un)support the metacognitive processes involved in mathematical problem-solving. The information allows teachers to spend sufficient time with particular types of mathematics problems whose solutions is determined by activation of metacognitive processes. This demonstrates the importance of including the activities for development of metacognitive monitoring in mathematics education.

## Introduction

The level of metacognitive knowledge in relation to achievement in mathematics was investigated in primary and secondary students (Desoete et al., 2001; Montague, 2008), adolescents (Kramarski and Mevarech, 2003), gifted students (Risemberg and Zimmerman, 1992; Swanson, 1992), and students with special needs (Grizzle-Martin, 2014; Lai et al., 2015). Several studies have shown the positive impact of metacognitive knowledge and metacognitive regulation on the students' achievement in mathematics (Swanson, 1990; González Castro et al., 2014) or mathematical problem solving (Schoenfeld, 1992). Yet, the training aimed at metacognition is not a usual practice in all primary schools in the Czech Republic. Since 2005, the schools have been allowed to vary the structure of their curriculum in grades (FEP, 2021), i.e., subsidize weekly lessons or group selected subjects into the one whole (e.g., science comprising chemistry, geography, biology, and physics). Commonly, schools do not use this option and design their curriculum in a more traditional way, while others are committed to innovative or alternative concepts, including respective activities focused on the development of metacognitive monitoring and metacognitive teaching. In this study, we do not discuss the definition of participating schools as “alternative” or “traditional.” The terminological inconsistency in viewing depends on the point of view of the relevant issues, including the policies and socio-economic issues (Prucha, 2012). We reflect the pedagogical point of view, where the term “alternative schools” means such institutions where they apply those educational approaches that differ in the content and form from the “traditional” approach, e.g., Montessori, Jena, Dalton, Decroly, Freinet, and Waldorf schools. Regardless of it, as to whether a school adheres to a traditional or alternative concept, the quality of the educational process depends on the teacher rather than on the affiliation of the school institution as such (Kasíková, 1994).

This study raises the question of how the individual aspects of self-regulated learning rehabilitate the pupils' mathematical performance at the end of the ISCED level 1 (5th grade) in schools with a traditional or alternative approach.

According to a review by Puustinen and Pulkkinen (2001), it is possible to divide all the models of self-regulated learning into the following two categories: goal-oriented and metacognitively oriented. In general, these models assume the existence of four phases: planning (preparatory phase), monitoring, control, and reaction-reflection involved in these 4 areas: cognition, motivation, behavior, and context. Therefore, there is the so-called double processing of information. First, at the level of the person, we include all internal prerequisites to achieve completion of the task as such (e.g., personality traits, metacognition, self-efficacy). Subsequently, the interaction between the task and the person takes place through the metacognitive processes regulating just the cognitive processes, and at the same time, there is the regulation and monitoring of affective and motivational processes. Thus, the processes interact with each other completely, resulting in the individual awareness and subsequent self-regulation of the learning process (Efklides, 2011, 2019; Panadero, 2017).

As several meta-analyses (Dignath et al., 2008; de Boer et al., 2018; Muncer et al., 2022) demonstrate the link between the various aspects of self-regulated learning and mathematical performance, we were interested in how the aspects of self-regulated learning of Grade 5 pupils related to their mathematical achievement.

## Theoretical background

### Metacognition

Metacognition can be understood as the ability to think about knowing oneself and the world around us, where the main purpose of this thinking is to improve one's own cognition. It is a conscious control and management of cognitive abilities. Interestingly, Cera et al. (2013) understand metacognition as the ability to control how we obtain information and how we further can process and store it in the mind. On the other hand, in this context, Winne (2018) sees thinking (not only thinking as a process, but also its properties) as the main interest of metacognition. According to Flavell (1979), metacognitive knowledge itself has a dual nature consisting of the knowledge of cognitive processes (metacognitive knowledge) and the knowledge that can be used to control cognitive processes (metacognitive regulation). While metacognitive regulation according to Flavell refers to the procedural components (goal setting, prediction, planning, application of strategies, self-questioning, organization, etc.), the metacognitive knowledge itself includes the knowledge and beliefs (entering the affective dimension) that an individual has about their cognitive resources (strategies, heuristics), the nature of the tasks, and the knowledge and beliefs about oneself and others as learning beings. This dual concept, despite some terminological inconsistencies, is still accepted across the professional discourse (Hong, 2020).

Several studies demonstrated the correlation between the ability to solve mathematical problems and both components of metacognition from grade one (Vo et al., 2014; Cornoldi et al., 2015) to grade three (Veenman et al., 2005; Van der Stel et al., 2010).

### Metacognitive knowledge

According to Flavell and Wellman (1975), metacognitive knowledge means an explicit content of long-term memory. It is a more static component of metacognition, being activated before the start of any cognitive enterprise (off-line). In the current literature, there is a division of metacognitive knowledge into the three areas (McCormick, 2003; Winne and Azevedo, 2014): declarative knowledge (any knowledge about oneself and factors influencing one's own cognition; knowledge about characteristic task situations; knowledge of strategies); procedural knowledge (knowledge referring to the way the task situation is performed); and, conditional knowledge (a knowledge of when, why and under what conditions it is appropriate to deploy a determined strategy). Borkowski et al. (2000) define relational metacognitive knowledge as well. Then, metacognitive knowledge is closely related to the specific field-domain knowledge. As stated by Veenman et al. (2006), metacognitive knowledge is built based on the acquisition of experiences and knowledge in a certain area, that over time makes it practically unrealistic to have an adequate metacognitive knowledge in a certain area without the individual having attained their own domain-specific knowledge. Therefore, the importance of metacognitive knowledge lies in the initiation of cognitive activities, meaning, how an individual uses them through the information processing and behavior in general. Although some results indicate a direct rate of development of declarative (Schneider, 2008) or relational and contextual metacognitive knowledge with age (Artelt and Neuenhaus, 2010), for example, in the case of the development of procedural knowledge, this ratio is not entirely clear. On the other side, declarative knowledge has been improved since the beginning of basic education, but the intensity of improvement decreases with age (Schneider and Löffler, 2016). Similarly, Annevirta et al. (2007) in their longitudinal study focused on kindergarten children to the first years of primary school pupils concluded that entering into school leads to an accelerated development of metacognitive knowledge. However, what types of mechanism lead to the described increase directly remained secret.

In recent years, other alternative approaches (Drigas and Mitsea, 2020, 2021) have emerged to relate this concept to the ordinary functioning of the individual in the 21st century to a view of metacognition that incorporates both already highly regarded characteristics of the metacognition construct (e.g., distinction between cognition and metacognition, self-monitoring, self-regulation), but also expanding and integrating the view of metacognition to include new aspects such as findings from cognitive neuroscience or the emphasis on the affective component. This approach is conventional with the view emerging in the late 1980s emphasizing the hot nature of cognition, i.e., that an individual's reasoning is influenced by their affective dimension (Brand, 1985).

In the field of mathematics education, Carr (2010) emphasizes the development of declarative knowledge for the development of conceptual knowledge. He claims that the quality of this knowledge supports the procedural knowledge and strategy building. Schneider and Artelt (2010) summarize the findings of several studies, stating that “metacognitive knowledge … predict mathematics performance in primary and secondary school settings even after differences in intellectual abilities have been taken into account” (pp. 158–159).

### Metacognitive regulation

Although metacognitive knowledge is an important prerequisite to assessing the real complexity of the task-situation and selecting appropriate strategies for a specific learning situation (Ríčan and Chytrý, 2020), optimization of this process can occur only when the strategy is successfully applied, i.e., when the knowledge of strategy is translated into a strategic learning behavior. Usually, this component of metacognition is divided into the three following areas (McCormick, 2003; Winne and Azevedo, 2014): planning (choosing goals and strategies before starting the work on the task); monitoring (conscious understanding of the task during its execution, including continuous monitoring through the self-instruction, self-testing and self-questioning); and evaluation (revision and control of the processes and products after the end of the activity), while in the empirical part of this work we focus on the aspects of metacognitive monitoring, because, in agreement with other authors (Winne and Hadwin, 1998), we find the construct as the central core of metacognition.

Metacognitive monitoring expresses a degree of agreement between the self-assessment of one's own learning, problem-solving, reading and memory performances, and the proven performance. Thus, it is a specific way the internal assessment of a task performance (Ackerman and Thompson, 2017) can be obtained in the course of different moments in the learning process (before, during, and after) and therefore associated with the different cognitive processes (Leonesio and Nelson, 1990). Regardless of the moment (before, during, after), just the metacognitive monitoring provides the student with their internal feedback on their personal learning progress, thus enabling the adaptation of learning processes as such. We refer to the assessment act as *metacognitive judgments* (Nelson, 1996; Dunn, 2004), that can be considered as an indicator of the level of metacognitive monitoring, distinguishing between the judgments of learning and post-dictions (confidence judgments). These are the two distinct cognitive processes (Metcalfe and Dunlosky, 2009), since the monitoring capabilities are divided on the basis of the three learning phases: (1) acquisition; (2) remembering; and (3) reproduction; where judgments of learning appear in the first phase and confidence judgments in the third phase (Nelson and Narens, 1990). Any personal performance prediction allows individuals to intervene in the learning mode by revealing some discrepancies between the current and desired target states and is therefore more relevant to an outcome of the current learning than post-prediction, that is primarily an assessment taking place at a time when the learning process has already ended, and its formative function points to the future (feedback, reflection, and lessons). In the empirical part of the work, we use confidence judgments, because it is not about the regulation of the ongoing learning process, but about the evaluation of the completed learning process. At the same time, post-diction is a much more accurate act than prediction (Hacker et al., 2000), and what we find more relevant for the purposes of this study.

The degree of certainty that one's own solution is correct may relate to either an overall estimate, i.e., overall score in the test, or a local estimate, i.e., correct solution to a particular problem. Although empirical studies suggest that the global estimate is more accurate and consistent than the local estimate (Schraw, 1994; Nietfeld et al., 2005), we find, based on the apparent differences in the accuracy of the local estimate between the more and less able students (Ibid), there is a higher expressing value in the context of taking into account the types and complexity of test items, including formative potential (specific identification of non-problematic items), in our opinion. Confidence-based data can be used to identify the 5 indices (Schraw, 2009) that refer to other dimensions of metacognitive monitoring, and there is no one-size-fits-all dominant method (Nietfeld et al., 2006). In the empirical part of this work, we focus on the *discrimination index* evaluating the degree within an individual can distinguish between confidence judgments for their correct answers and confidence judgments for their incorrect answers. Only in the first years of the primary education some pupils indicated a higher degree of certainty for correctly answered items than of certainty for incorrectly answered items (Pressley et al., 1987). The results in the 7- and 9-year-olds demonstrate this finding (Roderer and Roebers, 2010). While there were significant differences between the age groups in the confidence judgments area for the correct and incorrect answers, no differences appeared when only the confidence judgments for the correct answers were analyzed. That is why sometimes only the confidence judgments value for the incorrectly answered items is used as an indicator of the level of metacognitive monitoring (von der Linden and Roebers, 2006; Roebers et al., 2014).

### Motivation

The motivation theory and related empirical research suggest that just motivation plays an important role in the students' learning and academic performance at school (Wigfield and Cambria, 2010). Numerous researchers have studied the actual relationship between motivation and performance at school (Soenens and Vansteenkiste, 2005).

Certainly, elements such as engagement, motivation, and academic achievement are vital aspects in academic research as such. Interestingly, evidence suggests that the student involvement and motivation are positively associated with the improved quality of learning (Mohamed Mohamed Bayoumy and Alsayed, 2021). Such students who are truly motivated can see learning as an opportunity to fulfill their own curiosity and eagerness for knowledge (Rose, 2011). From this point of view, it is assumed that motivation in a certain area can lead to an increased involvement in related tasks. Therefore, a greater engagement is expected to lead to success gains on various measures (Skinner and Pitzer, 2012). In this context, it should be noted that such students generally have a complex relationship with mathematics as such (Mean and Maciejewski, 2021). This is due to the fact that they often do not see the relationship between learning mathematics and real life (Akbuga and Havan, 2021; Hecht et al., 2021). Research shows that students' motivation is to some extent related to a specific domain or subject in school (Hornstra et al., 2016). It has been found that the strength of the relationship between motivation and success varies across disciplines (Hornstra et al., 2016). These differences may not necessarily be due to the nature of the subjects themselves but may also be due to the students' assessment of the subjects. This statement is closely related to anxiety, where considerable research, for example, shows a negative association between mathematical anxiety and motivation for mathematics (Namkung et al., 2019; Zhang et al., 2019; Li et al., 2021).

Various studies have emphasized the importance of motivation in the field of cognitive performance as well as its relationship to metacognitive development (Lara Nieto-Marquez et al., 2021). Although it is recognized that the poor academic skills and math problems pose a risk to the development of school motivation (Klauda and Guthrie, 2015), studies on school motivation in children and young people with low math or reading skills are still rare (Parhiala et al., 2018).

### Self-efficacy

The concept of self-efficacy was first introduced by Bandura (1977) in an effort to elucidate changes in human behavior, defining the construct as a belief in one's own ability to organize and carry out activities to achieve set goals. Bandura (1994, 2011) also defined the four main ways in which self-efficacy affects human functioning (cognitive, motivational, emotional, and selective processes). Chan and Lam (2010) mention the two basic aspects of self-efficacy, namely (i) self-expectation (an individuals' belief in their own ability to take the necessary steps to achieve the desired result) and, (ii) expectation of the result (belief that a particular action or behavior will lead necessarily to the desired result). According to some authors (Williams and Rhodes, 2016), self-efficacy can be considered as a focal point, especially due to its strong predictive abilities. Together with the hope and optimism, self-efficacy is thus associated with some expectations about achieving future positive states (Feldman and Kubota, 2015; Unrau et al., 2018). Bandura (2011) points out that a high level of self-efficacy is manifested in the person's fearlessness in overcoming obstacles, setting higher goals, and persevering to achieve them (Pajares and Graham, 1999; Schunk and Meece, 2006). If such persons fail, then they do not give up and, on the contrary, rather, add to their effort, for example by acquiring new skills to meet the set goal. On the other hand, numerous individuals with a low level of self-efficacy are convinced that they are not able to achieve the set goals and so they do not even strive to do so. There are four main sources for this purpose (*mastery experiences, social modeling, social persuasion*, and *physical improvement*). In this regard, the whole feedback system is important, providing information about the evaluation of individuals' performances and affecting academic self-efficacy and self-regulation of learning (Brown et al., 2016). Cera et al. (2013) link the high levels of self-efficacy to the high levels of metacognitive abilities, with negative learning experiences underlying the low self-efficacy, critical thinking, and metacognitive involvement.

Several authors have already tried to define mathematical self-efficacy. Burnham (2011, p. 4 in Smetáčková and Vozková, 2016) speaks of it as an individual's confidence in their ability to perform successfully in mathematics. Betz and Hackett (1983 in Pajares, 2005, p. 300) add that it is “*individuals' judgments of their capabilities to solve specific mathematics problems, perform mathematics-related tasks, and succeed in mathematics-related courses*.” The predictive potential of self-efficacy in the context of mathematical achievement is well-documented (Coutinho and Neuman, 2008; Chang, 2015).

## Methodology

Garofalo and Lester (1985) stressed that the description and analysis of cognitive strategies alone was not enough to understand problem solving and highlighted metacognitive processes. They considered including the metacognitive activities described in their framework (entitled *Cognitive-Metacognitive Framework for Studying Mathematical Performance*) the most useful form of introducing these processes into teaching. Their problem-solving framework comprised the four activities present in solving any mathematical task: *orientation, organization, execution*, and *verification*, while the individual steps perceived from a theoretical point of view correspond to the various aspects of metacognition activation. The importance of this classification derives from the fact that any metacognitive actions are more likely to occur in one phase than in another, depending on the nature of the mathematical task. In order for the students themselves to master this general procedure, it is necessary to pay strongly-focused attention to the application of this model in different subjects as well; the use of various mathematical tasks, and the use of various teaching techniques embedded in the framework of teaching metacognition is not for mathematics alone.

### Metacognitive knowledge

The MAESTRA 5-6+ tool is based on the qualitative standard emphasizing the relative adequacy of the chosen strategy in the context of task specificity, opposed to the quantitative standard emphasizing the frequency of using the strategies (Wirth and Leutner, 2008). Specificity of the strategies can be achieved through increasing the specificity of tasks (Leopold and Leutner, 2002; Samuelstuen and Bråten, 2007). Therefore, questionnaires investigating the frequency of use of various strategies often fail to predict the students' performance. Clearly, the correlation questionnaires measuring the frequency of use of school-based strategies are negligible (Sperling et al., 2002; Lind and Sandmann, 2003; Cromley and Azevedo, 2006). The tool MAESTRA 5-6+ requires an answer (1) what strategy will somebody use (declarative knowledge); (2) in relation to other disposables (relational knowledge), but also (3) when/under what conditions (conditional knowledge) will it apply in the context of understanding the characteristics of the described, and (4) task situation (declarative knowledge)? Therefore, any real deficit in any of the above areas may lead to erroneous strategic evaluation (Artelt and Neuenhaus, 2010; Neuenhaus, 2011). The tool contains the 5 specific mathematical scenarios that correspond to the framework model of the four phases of cognitive activity in solving mathematical problems (Polya, 1957): (i) understanding the task assignment (understanding the problem and defining it), (ii) planning the individual solution steps (design of solution strategies), (iii) implementation of the plan (implementation of strategies), and (iv) evaluation and reflection of the solution (looking back to verify the conclusions, checking the results). Polya (1957) assigned the cognitive strategies leading to the goal of the current phase of processing the problem for each step, accepting the problem-solving non-linearity. Later, Garofalo and Lester (1985) added the metacognitive strategies in the end.

The five or six different strategic alternatives varying in functionality and efficiency are assigned to the five specific mathematical scenarios. Thus, pupils themselves can evaluate the effectiveness of the alternative regarding the quality and adequacy not only in relation to the submitted task scenario, but also in relation to other offered alternatives on a six-point scale. As a comparative measure for the respondents' judgments (projection of their own experience about strategies and conditions of their use), expert opinions were obtained within the content validation of the tool construct (Chytrý et al., 2014). If the statement is in accordance with a predetermined expert opinion, the comparison of the pair is considered correct (1), unless it is inconsistent as incorrect (0). The criterion limit was set at 80 %, i.e., that at least the 4 out of 5 experts had to agree that, for example, in scenario 1, strategic alternative *a* *is* more appropriate than another strategic alternative *b* (*a*> *b*). The criteria boundaries and selectivity then led to the expected reduction in the number of pairwise comparisons. Finally, there are a total of the 34 comparisons and the participant can score 0–34 points.

### Self-efficacy and motivation

The authors (Smetáčková and Vozková, 2016) of the Czech version of the self-efficacy mapping tool standardized and constructed according to the Bandura recommendations (Bandura, 1997), stated the reliability of the tool α = 0.953 (α = 0.72 in the presented research). The self-efficacy mapping tool contains the thirty items, answered using a five-point Likert scale (Likert, 1932). The motivation tool (α = 0.922) comprises the six-items, and also uses a five-point Likert scale. For both tools, it was not possible to discuss the issue of an even or odd number of scales, as stated by Rod (2012), as they were already piloted in the Czech environment. The tools are evaluated through the sum of responses for all items (ordinal data). In this case (sum) we will proceed from the conclusions and the way of using the given scale by a number of authors (Maurer and Pierce, 1998; Vickers, 1999) and consider the given variable as an interval. Boone and Boone (2012) add that the parametric statistical methods need to be used when processing data on an interval scale. As the part of self-efficacy, the participant can obtain 30–150 points. The lower the final score, the higher the level of mathematical self-efficacy. In case of motivation, the scale is 6–30 points. It is true that the lower the value obtained, the worse the student's relationship is to mathematics.

### Math achievement

The tool was created by combining the two different tools tested in CERMAT (a company designing the national testing) research, MA2ACZZ506DT (test A) and M5PZD15C0T01 (test B). The renumbering of individual items and their adjustment is described in detail in Chytrý et al. (2020). To verify the content validity, expert opinions were considered. We obtained the feedback from six experts, mathematics didacticians, fulfilling the requirements defined by Ríčan and Chytrý (2016) based on the work of Neuenhaus (2011). The expert was understood as a person demonstrating their direct relationship to the strategic learning in context of reading comprehension (university teacher of the subject; finished PhD degree in relevant field). We have adapted and partially modified this approach in our survey. In addition to the Neuenhaus's analogous approach, we also addressed the people who published at least 3 relevant articles related to strategic learning in the last 5 years. The Bloom's revised cognitive goal taxonomy by Krathwohl and Anderson (2010) was used to classify questions according to cognitive demands. The classification of the task as a lower or higher cognitively intensive category was specified when at least four of the six experts agreed on the assignment. In total, 83% of the items correspond to the higher cognitive demands, particularly important due to metacognitive monitoring. The responses were evaluated as 0 (incorrect), 1 (correct) or NA (missing). The arithmetic mean of the measured values is then a suitable point estimate of the parameter *p* of the alternative distribution, the probability that the randomly selected student will answer the question correctly. According to Chráska (1999), the Difficulty Index was calculated according to the relation $p\;=\;\frac{x_{s}}{x}\;$and the difficulty value then *q* = 1−*p*. The suitable tasks are included in the interval *p*∈〈0.20;0.80〉, and the tasks with *p* < 0.20 or *p*>0.80 are considered suspicious. In this study there were only the 4 tasks considered suspicious tasks. The instrument itself shows the high reliability examined based on Kudera–Richardson Formula 21, *KR*_21_ = 0.807 (split half with the division into the even and odd items 0.771 and after correction by Spearman-Brown formula 0.871). Thus, the values meet the required properties (Tavakol and Dennick, 2011). Moreover, sensitivity was solved using a point biserial sensitivity coefficient. Compared to our previous study (Chytrý et al., 2020), one item was excluded, due to the incorrect use of confidence scales by pupils. Pupils could achieve 0–20 points in the final version of the math achievement test.

### Metacognitive monitoring

Each item was assigned by a difficulty scale, a line where pupils marked how confident they were that they had answered the mathematics question correctly. In the context of determining the level of metacognitive monitoring, we used the so-called discrimination index *D*_*i*_ through the confidence judgments that can be calculated according to the formula.


(1) $$D_{i}=\frac{1}{N}\left[\sum_{i=1}^{N_{c}}c_{icorrect}-\sum_{i=1}^{N_{i}}c_{iincorrect}\right]$$

Where *N*_*c*_ corresponds to the frequency of correctly answered items, *N*_*i*_ is the frequency of incorrectly answered items, *c*_*icorrect*_ corresponds to the degree of certainty for correctly answered and *c*_*iincorrect*_ to the degree of certainty for incorrectly answered items and *N* is the overall number of items (correctly and incorrectly answered). Any positive value of discrimination index indicates that the individual is more confident in the correctly answered items than in incorrectly answered items. The positive value of discrimination index can be interpreted as *metacognitive awareness of the* correct performance, as the respondent provided greater certainty for the correctly answered items (compared to the incorrectly answered items). Conversely, a negative value indicates that the individual is more confident in the incorrectly answered items than in correctly answered items (Dougherty and Sprenger, 2006).

### Sampling and data collection

As the first part of data collection, we selected those schools in the Czech Republic where mathematics is taught by the genetic constructivism approach, in the Czech and Slovak Republics known as the Hejný's method (Artigue et al., 2020). Respondents from these types of schools were selected based on multi-stage random sampling, so that we first randomly selected a school and then a class in it. The basic unit of research sample selection was not individual students, but entire school classes. Subsequently, the collection at other types of schools had to be regulated by certain predetermined criteria so that the proportionality, social status (this part was mapped based on an interview with the teacher), resulted in equal conditions for each of the respondents to the greatest possible extent. The given proportionality lies in the fact that each of the four types of schools (traditional schools; schools teaching mathematics using genetic constructivism, i.e., the Hejný method; Montessori schools; and Dalton schools) were always from one place (village, city, region); e.g., if the school where mathematics is taught with the approach of genetic constructivism is a school of small size and village type, there were also other types of schools of similar size and from the village, town, or city located closest to the original school.

The data collection took place in June 2021 (Tuesday to Friday). The tools were not distributed during the first lesson of the school-day, the regular teaching unit was inserted between the first and second lessons of data collection, and during the data collection day the pupils were not evaluated in any way. The basic unit of selection of the research sample was not the individual pupils, but the entire school classes. The most important information and instructions included: (i) Sequence of distribution of individual tools, namely the metacognitive test of mathematical knowledge, mathematical self-efficacy (completion of these tools took place in one lesson), as well as the didactic test in mathematics (completion of this tool took place in the second lesson); (ii) Transmission of information on how the individual instruments are completed; (iii) Definition of time requirements, where the time for individual tests was defined as 20–25, 20, and 40–45 min, respectively.

From the total of 1,133 respondents, students of Grade 5, only the responses without any missing values were analyzed. We analyzed the data from 36 schools out of which: (i) 179 pupils were educated according to the Hejný's method, (ii) 292 pupils from an ordinary primary school, and (iii) 177 pupils completing the Dalton teaching plan. After all responses with missing values were removed, data from 648 respondents were analyzed.

## Results

**Figure 1:**
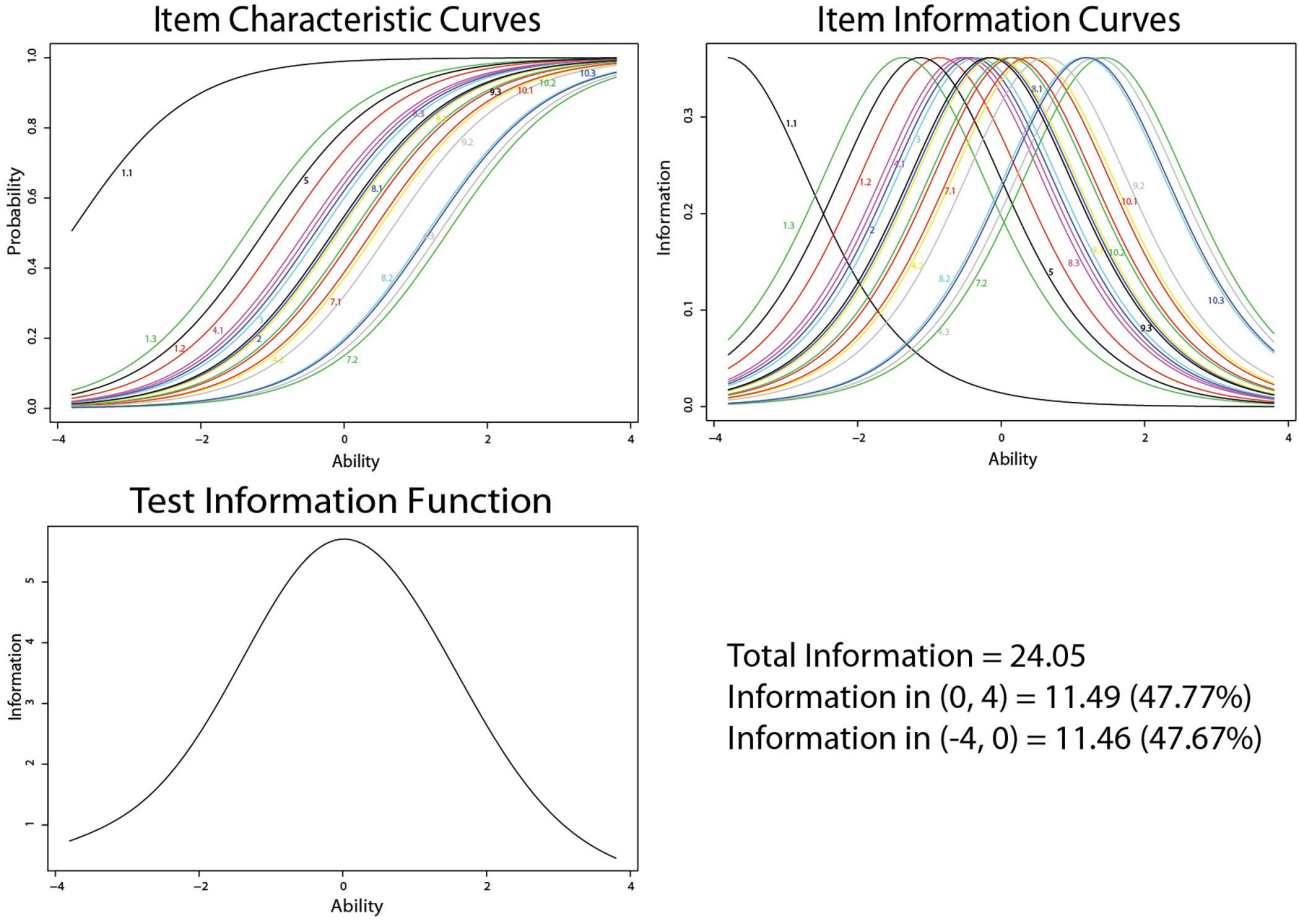
Item characteristics of mathematical achievement test.

The descriptive analysis of the sample is summarized in Table 1. The item characteristic curves (ICC), item information curves (IIC) for all items, and test information for the test of mathematics achievement are in Figure 1. In our data, all the ICCs for the test items have an “S” -shaped curve, illustrating that their degrees of difficulty and discrimination vary within a reasonable fit range. Most of the IICs for the test items show a broad coverage of students' abilities. The test information curve suggests that the test of mathematics achievement that was used provided almost the same information on students with the higher abilities as on students with the lower abilities.

**Table 1:** Descriptive analysis according to the preferred learning management strategies at different types of schools.

	**Hejný**				**Ordinary school**				**Dalton**			
	***X*_1_**	***X*_2_**	***X*_3_**	***X*_4_**	***X*_1_**	***X*_2_**	***X*_3_**	***X*_4_**	***X*_1_**	***X*_2_**	***X*_3_**	***X*_4_**
Average	21.00	12.00	60.50	0.20	20.00	12.00	61.00	0.18	19.48	12.38	66.10	0.17
Median	26.00	10.00	50.00	0.40	22.00	15.00	64.00	0.90	20.00	12.00	63.50	0.15
Mode	4.96	5.21	17.00	0.26	5.26	5.24	18.61	0.36	21.00	12.00	58.00	−0.28
SD	30.00	26.00	109.00	0.90	30.00	29.00	138.00	1.00	5.38	6.14	18.19	0.31
Max	8.00	2.00	33.00	−0.39	6.00	0.00	30.00	−0.83	30.00	28.00	116.00	0.90
Min	21.00	12.00	60.50	0.20	20.00	12.00	61.00	0.18	0.00	0.00	31.00	−0.48

*X*_1_, Relation to mathematics; *X*_2_, Metacognitive knowledge; *X*_3_, Self-efficacy; *X*_4_, Discrimination index.

The regression analysis was performed by the ENTER method, that first addressed the issue of detection of outliers, normality of residues, testing of homoscedasticity, and multicollinearity. The aim was to determine an influence of the independent variables: (i) Pupil's relation to mathematics; (ii) Metacognitive knowledge; (iii) Self-efficacy; and (iv) Metacognitive monitoring focused on the achievement in mathematics (dependent variable). Those independent variables for which the partial *t*-tests were insignificant were gradually excluded from the model and subsequently the parameters of the resulting model were estimated.

The table shows that the models will differ for each preferred learning management strategy. Given the parameters (Table 2), the model is considered suitable to capture the variability of the variable *y* for all cases. The shapes of the respective regression equations are then as follows:

**Table 2:** Chosen parameters of the regression model.

	**Insert all variables at once**						**After removing insignificant variables**					
	**Hejný**		**Ordinary elementary school**		**Dalton**		**Hejný**		**Ordinary elementary school**		**Dalton**	
	***R** = **0.931;***		***R** = **0.953;***		***R** = **0.949;***		***R** = **0.929;***		***R** = **0.953;***		***R** = **0.948;***	
	***R**^**2**^ = **86.290%***		***R**^**2**^ = **90.756%***		***R**^**2**^ = **89.749%***		***R**^**2**^ = **86.338%***		***R**^**2**^ = **90.799%***		***R**^**2**^ = **89.808%***	
	***F** = **288.9;***		***F** = **717.7;***		***F** = **397.2;***		***F** = **579.2;***		***F** = **1441.9;***		***F** = **798.5;***	
	***p**<**0.001***		***p**<**0.001***		***p**<**0.001***		***p**<**0.001***		***p**<**0.001***		***p**<**0.001***	
	***Coef***.	***P-val***.	***Coef***.	***P-val***.	***Coef***.	***P-val***.	***Coef***.	***P-val***.	***Coef***.	***P val***.	***Coef***.	***P-val***.
TO	7.91	0.00***	7.77	0.00***	10.4	0.00***	8.19	< 0.01***	7.39	< 0.00***	9.83	< 0.001***
*X*_1_	0.02	0.40	0.04	0.09*	−0.02	0.39	–	–	0.05	0.006***	–	–
*X*_2_	0.03	0.05 *	0.00	0.63	0.01	0.57	0.04	0.035	–	–	–	–
*X*_3_	−0.02	0.83	0.00	0.50	−0.02	< 01***	–	–	–	–	−0.02	< 0.001***
*X*_4_	12.9	0.00***	12.4	0.00***	12.9	0.00***	13.01	< 0.00***	12.55	< 0.00***	12.99	< 0.001***

Regression analysis according to the preferred learning management strategies at different types of schools (all variables): ^*^ten percent level of significance, ^**^five percent level of significance, ^***^one percent level of significance.

*X*_1_, Relation to mathematics; *X*_2_, Metacognitive knowledge; *X*_3_, Self-efficacy; *X*_4_, Discrimination index.

Hejný: *y* = 8.196+ 0.041·*X*_2_+13.010·*X*_4_Ordinary elementary school: *y* = 7.388 + 0.050·*X*_1_+ 12.553·*X*_4_Dalton: *y* = 9.837 − 0.023·*X*_3_+12.992·*X*_4_

The analysis continued assuming that the items are divided into the lower and higher complexity of the items of a mathematical achievement test according to the Rasch's model. The reliability value for items of the higher difficulty is equal to 0.751 for (split half) and 0.727 for *KR*_20_. In the case of items of the lower cognitive intensity, the reliability value is then 0.723 (split half) and 0.716 for *KR*_20_. Further analyses are divided into several sections, namely, the same models, but with the difference that the items are divided into the lower and higher difficulties (Table 3).

**Table 3:** Regression analysis according to the preferred learning management strategies at different types of schools (all variables).

	**Items of the higher cognitive intensity**											
	**Inserting all variables**						**After removing insignificant variables**					
	**Flock**		**Ordinary elementary school**		**Dalton**		**Flock**		**Ordinary elementary school**		**Dalton**	
	***R** = **0.907;***		***R** = **0.932;***		***R** = **0.911;***		***R** = **0.907;***		***R** = **0.932;***		***R** = **0.910;***	
	***R** ^2^ = **82.015%***		***R** ^2^ = **86.832%***		***R** ^2^ = **82.727%***		***R** ^2^ = **82.001%***		***R** ^2^ = **86.832%***		***R** ^2^ = **82.629%***	
	***F** = **209.626;***		***F** = **482.4;***		***F** = **217.7;***		***F** = **279.011;***		***F** = **482.4;***		***F** = **431.489;***	
	***p**<**0.001***		***p**<**0.001***		***p**<**0.001***		***p**<**0.001***		***p**<**0.001***		***p**<**0.001***	
	***Coef***.	***P-val***.	***Coef***.	***P-val***.	***Coef***.	***P-val***.	***Coef***.	***P-val***.	***Coef***.	***P-val***.	***Coef***.	***P-val***.
TO	3.25	< 0.01***	2.65	< 0.01***	4.19	< 0.01***	3.76	< 0.01***	2.65	< 0.01***	4.35	< 0.01***
*X*_1_	0.02	0.299	0.04	< 0.01***	−0.01	0.80	–	–	0.04	< 0.01***	–	–
*X*_2_	0.02	0.062*	0.02	0.05*	0.02	0.09*	0.03	0.04	0.02	0.05*	–	–
*X*_3_	−0.01	0.055*	−0.01	0.02**	−0.02	< 0.01***	−0.01	< 0.01***	−0.01	0.02**	−0.02	< 0.01***
*X*_4_	5.12	< 0.01***	4.83	< 0.01***	4.91	< 0.01***	5.13	< 0.01***	4.83	< 0.01***	5.02	< 0.01***
	**Items of the lower cognitive intensity**											
	**Inserting all variables**						**After removing insignificant variables**					
	**Flock**		**Ordinary elementary school**		**Dalton**		**Flock**		**Ordinary elementary school**		**Dalton**	
	***R** = **0.913;***		***R** = **0.942;***		***R** = **0.950;***		***R** = **0.911;***		***R** = **0.941;***		***R** = **0.950;***	
	***R** ^2^ = **82.999%***		***R** ^2^ = **88.496%***		***R** ^2^ = **90.104%***		***R** ^2^ = **82.981%***		***R** ^2^ = **88.585%***		***R** ^2^ = **90.294%***	
	***F** = **224.4;***		***F** = **562.5;***		***F** = **413.0;***		***F** = **893.326;***		***F** = **2267.02;***		***F** = **832.0;***	
	***p**<**0.001***		***p**<**0.001***		***p**<**0.001***		***p**<**0.001***		***p**<**0.001***		***p**<**0.001***	
	***Coef***.	***P-val***.	***Coef***.	***P-val***.	***Coef***.	***P-val***.	***Coef***.	***P-val***.	***Coef***.	***P-val***.	***Coef***.	***P-val***.
TO	4.97	0.00***	5.08	0.00***	5.99	0.00***	5.68	< 0.01***	5.42	< 0.01***	5.62	< 0.01***
*X*_1_	0.01	0.64	0.01	0.42	−0.01	0.49	–	–	–	–	–	–
*X*_2_	0.02	0.17	0.01	0.92	−0.01	0.87	–	–	–	–	–	–
*X*_3_	0.01	0.26	0.01	0.72	−0.01	0.047**	–	–	–	–	−0.01	0.045
*X*_4_	7.04	0.00***	6.99	0.00 ***	768	0.00***	7.02	< 0.01***	7.04	< 0.01***	7.64	< 0.01***

^*^10% level of significance, ^**^5% level of significance, ^***^1% level of significance.

*X*_1_, Relation to mathematics; *X*_2_, Metacognitive knowledge; *X*_3_, Self-efficacy; *X*_4_, Discrimination index.

As in the case of the first table, the models will differ for each of the preferred learning management strategies. The shapes of the respective regression equations are then as follows:

Items of the higher cognitive intensityHejný: *y* = 8.196 + 0.03·*X*_2_−0.01*X*_3_+ 5.13·*X*_4_Ordinary elementary school: *y* = 2.65 + 0.04·*X*_1_+0.02·*X*_2_−0.01·*X*_3_+ 4.83·*X*_4_Dalton: *y* = 4.35 − 0.02·*X*_3_+5.02·*X*_4_Items of the lower cognitive intensityHejný: *y* = 5.68 +7.02·*X*_4_Ordinary elementary school: *y* = 5.42+7.04·*X*_4_Dalton: *y* = 5.62 − 0.01·*X*_3_+7.64·*X*_4_

The subsequent differences were even more detailed in the sense that the students themselves were divided according to their performance on the mathematical test (Table 4).

**Table 4:** Regression analysis according to the preferred learning management strategies at different types of schools (all variables).

	**Low-achieving students**											
	**Inserted all variables**						**After removing insignificant variables**					
	**The test as a whole**		**Challenging tasks**		**Less demanding**		**The test as a whole**		**Challenging tasks**		**Less demanding**	
	***R** = **0.609;***		***R** = **0.476;***		***R** = **0.784;***		***R** = **0.608;***		***R** = **0.469;***		***R** = **0.782;***	
	***R** ^2^ = **35.596%***		***R** ^2^ = **20.727%***		***R** ^2^ = **60.479%***		***R** ^2^ = **37.022%***		***R** ^2^ = **22.225%***		***R** ^2^ = **60.749%***	
	***F** = **23.246;***		***F** = **11.523;***		***F** = **62.594;***		***F** = **46.735;***		***F** = **44.378;***		***F** = **125.778;***	
	***p**<**0.001***		***p**<**0.001***		***p**<**0.001***		***p**<**0.001***		***p**<**0.001***		***p**<**0.001***	
	***Coef***.	***P-val***.	***Coef***.	***P-val***.	***Coef***.	***P-val***.	***Coef***.	***P-val***.	***Coef***.	***P-val***.	***Coef***.	***P-val***.
TO	734	0.00***	1.46	< 0.01***	5,931 th most common	< 0.00***	7.71	< 0.01***	1.26	< 0.01***	5.54	< 0.01***
*X*_1_	0.02	0.56	< 0.01	0.72	−0.01	0.62	–	–	–	–	–	–
*X*_2_	−0.01	0.71	– < 0.01	0.91	−0.01	0.51	–	–	–	–	–	–
*X*_3_	−0.02	0.03**	– < 0.01	0.36	−0.01	0.02**	−0.02	< 0.01***	–	–	−0.01	0.01
*X*_4_	6.98	0.00***	1,723rd most common	< 0.00***	6.38	< 0.00***	7.01	< 0.01***	1.63	< 0.01***	6.31	< 0.01***
	**High-achieving students**											
	**Inserted all variables**						**After removing insignificant variables**					
	**The test as a whole**		**Challenging tasks**		**Less demanding**		**The test as a whole**		**Challenging tasks**		**Less demanding**	
	***R** = **0.901;***		***R** = **0.939;***		***R** = **0.818;***		***R** = **0.900;***		***R** = **0.939;***		***R** = **0.817;***	
	***R** ^2^ = **80.95%***		***R** ^2^ = **88.08%***		***R** ^2^ = **73.98%***		***R** ^2^ = **81.03%***		***R** ^2^ = **88.22%***		***R** ^2^ = **66.52%***	
	***F** = **299.5;***		***F** = **519.8;***		***F** = **140.0;***		***F** = **595.9;***		***F** = **1044.2;***		***F** = **280.3;***	
	***p**<**0.001***		***p**<**0.001***		***p**<**0.001***		***p**<**0.001***		***p**<**0.001***		***p**<**0.001***	
	***Coef***.	***P-val***.	***Coef***.	***P-val***.	***Coef***.	***P-val***.	***Coef***.	***P-val***.	***Coef***.	***P-val***.	***Coef***.	***P-val***.
TO	9.90	< 0.00***	3.69	< 0.00***	6,264 th most common	< 0.00***	10.71	< 0.01***	3.76	< 0.01***	6.75	< 0.01***
*X*_1_	0.02	0.21	0.01	0.59	0.01	0.26	–	–	–	–	–	–
*X*_2_	0.02	0.024**	0.01	0.049**	0.01	0.47	0.02	0.02 **	0.01	0.042	–	–
*X*_3_	0.01	0.11	0.01	0.89	0.01	0.01 **	–	–	–	–	0.01	0.02
*X*_4_	8.89	< 0.00***	4.27	< 0.00***	0.86	< 0.00***	0.81	< 0.01***	4.28	< 0.01***	4.90	< 0.01***

^*^10% level of significance, ^**^5% level of significance, ^***^1% level of significance.

*X*_1_, Relation to mathematics; *X*_2_, Metacognitive knowledge; *X*_3_, Self-efficacy; *X*_4_, Discrimination index.

Less successful studentsHejný: *y* = 7.71 − 0.02·*X*_3_+7.02·*X*_4_Ordinary elementary school: *y* = 1.26+1.62·*X*_4_Dalton: *y* = 5.54 − 0.01·*X*_3_+6.31·*X*_4_More successful studentsHejný: *y* = 10.71+0.02·*X*_2_ +8.81·*X*_4_Ordinary elementary school: *y* = 3.76+0.01·*X*_2_+4.28·*X*_4_Dalton: *y* = 6.75+0.01·*X*_2_+4.90·*X*_4_

## Discussion

Regardless of the demands of the tasks, metacognitive monitoring saturated the performance in mathematics at the statistical level of significance *p* < 0.01. This finding is in partial contradiction with the research showing that the benefits of involving metacognitive processes occur primarily in the mid-range tasks (Prins et al., 2006). The results of this study also demonstrate that regardless of the type of school, the aspect of metacognitive monitoring is a strong determinant (*p* < 0.01) of mathematical achievement. Therefore, these findings underline the crucial role of metacognitive monitoring in the field of mathematics education and suggest that schools should look for different ways to implement, definitely and without delay, a metacognitive monitoring in their curricula.

Thus, metacognitive knowledge proved to be a significant predictor (*p* < 0.10) for the more challenging tasks across all types of schools. Interestingly, lower-achieving students did not benefit from activating metacognitive knowledge alone. In the case of higher-achieving pupils, metacognitive knowledge was significant (*p* < 0.05) in the case of more demanding tasks. On the other hand, Meneghetti et al. (2007) stated that there is a discrepancy between the knowledge of a strategy and appropriate choice of a strategy, especially for the lower-achieving pupils. It is possible that lower-achieving pupils in this study failed to choose a suitable strategy that would help them to solve mathematical problems of a higher difficulty. We recommend focusing on the aspects of metacognitive knowledge in the context of adequate choice of strategies especially for the less able pupils. As Rozencwajg (2003) states, teaching metacognitive strategies, especially for the less-achieving pupils, could be one way to improve their academic success.

On the other hand, the importance of self-efficacy varied depending on the difficulty of the item, the pupils' achievement in the mathematical test, and the type of school. While statistical significance showed a different degree of intensity across the individual schools (Hejný *p* < 0.10, ordinary primary school *p* < 0.05, Dalton *p* < 0.01), in the case of the lower intensity items, statistical significance only showed in pupils from the Dalton elementary schools (*p* < 0.05). The results of this study also show that, whether they are mainstream schools or schools enrolling in a specific educational program (Hejný, Dalton), the aspect of metacognitive monitoring is a strong determinant (*p* < 0.01) of mathematical performance. This finding underscores the crucial role of metacognitive monitoring, regardless of the non-specific curriculum or program the school subscribes to.

### Limitations

Even though the sampling was not and could not be purely random for the all the mentioned types of schools, it was managed according to certain predetermined criteria. The obtained sample contained the 36 schools from all regions of the Czech Republic and may be considered as representative. We also focused on the limits of the strongest predictor across the individual schools and the level of difficulty of items and confidence judgments as an indicator of metacognitive monitoring. The pupils' confidence judgments are strongly influenced by the (un)conscious heuristics, e.g., familiarity, fluency, font (Finn and Tauber, 2015) used when working with the given teaching material. The familiarity of the task in the context of the pupils' previous knowledge is a potential intervening variable increasing the value of confidence judgments (Metcalfe and Finn, 2008). Thus, the confidence judgments are not only the (cognitive) result of (non) present knowledge, but it is a pervasive concept that also includes factors such as feelings, metacognitive experiences, or epistemic beliefs alone. The findings of research by Miele et al. (2013) show that when students believe that the intelligence is an innate ability, they perceive some difficulties (reduced fluency) during the processing as an indicator that they are reaching their limits and the result is a lower confidence judgment in the end. On the other hand, students who believe that intelligence is a malleable factor usually do not interpret the processing of difficulties as the limits of their inner abilities at all (their confidence judgments do not tend to decline or fluctuate). For this reason, in the subsequent regression models, we recommend capturing other (mediating) variables (e.g., socio-economic status, prior knowledge, epistemic beliefs).

## Conclusions

The metacognitive monitoring alone is important to identify any difficulties in the learning process (and to respond appropriately by implementing a proper and useful learning strategy) and to adapt the available learning time to the learning requirements or to complete the learning process in time when it is successful or in current settings without prospects for a successful solution (Metcalfe and Dunlosky, 2009).

For the immediate educational reality, discrimination is an important indicator for the time of learning various parts of the curriculum. Discrimination thus creates a springboard for deciding (1) what is not yet sufficiently controlled (what passages of the curriculum and for how long they are to be studied) and, conversely, what already is controlled enough (it is effective to abandon the study of these passages) and at the same time, which questions in the test need to be returned to reveal which answers are correct and which need to “*cross out*” and start searching again in the memory or in the assigned text. Therefore, the pupils being aware of what they already know and do not know can spend a redundant amount of time on the passages they have already mastered and at the same time can pay an increased attention and amount of time to those sections they do not understand or do not remember thoroughly. “*[T]he monitoring process informs the learner about [their] learning progress and therefore builds a defining basis for their self-initiating learning behavior*.” (Roebers et al., 2014, p. 142). Thus, through the discrimination, we can gain information about what we know and do not know, and then we have access to designing respective and proper strategies and choosing between those who can help us to succeed. Rhodes and Kelley (2005) conclude that, as the part of completing a test, a follow-up process for incorrect answers is far more crucial to output than a follow-up process for correct answers.

## Data availability statement

The raw data supporting the conclusions of this article will be made available by the authors, without undue reservation.

## Ethics statement

The studies involving human participants were reviewed and approved by Ethics Committee of J. E. Purkyne University in Usti nad Labem Czech Republic. Written informed consent to participate in this study was provided by the participants' legal guardian/next of kin.

## Author contributions

All authors listed have made a substantial, direct, and intellectual contribution to the work and approved it for publication.

## Funding

This research was funded by the Slovak Research and Development Agency under the contract No. APVV-20-0599 and by the Scientific Grant Agency of the Ministry of Education, Science, Research and Sport of the Slovak Republic under contract No. KEGA 015UK F-4/2021. This research was also supported by two projects conducted at University of Jan Evangelista Purkyne in Ústí nad Labem, Czech Republic: UJEP-SGS-2021-43-003-2 and UJEP-SGS-2022-43-004-2.

## Conflict of interest

The authors declare that the research was conducted in the absence of any commercial or financial relationships that could be construed as a potential conflict of interest.

## Publisher's note

All claims expressed in this article are solely those of the authors and do not necessarily represent those of their affiliated organizations, or those of the publisher, the editors and the reviewers. Any product that may be evaluated in this article, or claim that may be made by its manufacturer, is not guaranteed or endorsed by the publisher.
